# Reduced levels of N’-methyl-2-pyridone-5-carboxamide and lysophosphatidylcholine 16:0 in the serum of patients with intrahepatic cholangiocarcinoma, and the correlation with recurrence-free survival

**DOI:** 10.18632/oncotarget.22607

**Published:** 2017-11-22

**Authors:** Kyung-Hee Kim, Jungnam Joo, Boram Park, Sang-Jae Park, Woo Jin Lee, Sung-Sik Han, Tae Hyun Kim, Eun Kyung Hong, Sang Myung Woo, Byong Chul Yoo

**Affiliations:** ^1^ Biomarker Branch, Research Institute, National Cancer Center, Goyang 10408, Republic of Korea; ^2^ Omics Core Laboratory, Research Institute, National Cancer Center, Goyang 10408, Republic of Korea; ^3^ Biometrics Research Branch, Research Institute, National Cancer Center, Goyang 10408, Republic of Korea; ^4^ Center for Liver Cancer, Hospital, National Cancer Center, Goyang 10408, Republic of Korea

**Keywords:** metabolic biomarkers, intrahepatic cholangiocarcinoma

## Abstract

We searched for metabolic biomarkers that may predict the prognosis of patients with intrahepatic cholangiocarcinoma (IHCC). To this end, a total of 237 serum samples were obtained from IHCC patients (*n* = 87) and healthy controls (*n* = 150), and serum metabolites were analyzed by liquid chromatography-tandem mass spectrometry (LC-MS/MS). Two stratified algorithms were used to select the metabolites, the levels of which predicted the prognosis of IHCC patients. We performed MS/MS and multiple-reaction-monitoring MS analyses to identify and quantify the selected metabolites. Continuous biomarker levels were dichotomized based on cutoffs that maximized between-group differences in recurrence-free survival (RFS) in terms of the log-rank test statistic. These RFS differences were analyzed using the log-rank test, and survival curves were drawn with the aid of the Kaplan–Meier method. Six metabolites (l-glutamine, lysophosphatidylcholine [LPC] 16:0, LPC 18:0, N’-methyl-2-pyridone-5-carboxamide [2PY], fibrinopeptide A [FPA] and uric acid) were identified as candidate metabolic biomarkers for predicting the prognosis of IHCC patients. Of these metabolites, levels of l-glutamine, uric acid, LPC 16:0, and LPC 18:0 were significantly lower in the serum from IHCC patients, whereas levels of 2PY and FPA were significantly higher (*p* < 0.01). 2PY and LPC 16:0 showed significantly better RFS at low level than high level (2PY, median RFS: 15.16 months vs. 5.90 months, *p* = 0.037; LPC 16:0, median RFS: 15.62 months vs. 9.83 months, *p* = 0.035). The findings of this study suggest that 2PY and LPC 16:0 identified by metabolome-based approaches may be useful biomarkers for IHCC patients.

## INTRODUCTION

The biliary tract and drainage system include the intra- and extra-hepatic bile ducts and gallbladder. “Cholangiocarcinoma” refers to a tumor arising from the bile duct epithelium. Intrahepatic cholangiocarcinoma (IHCC) is the second most common primary hepatobiliary cancer (after hepatocellular carcinoma). IHCCs are rare, difficult to diagnose, and have a poor prognosis [1]. Cholangiocarcinoma is generally rare in Western countries [2], but is more common in Korea, with about 3,500 cases diagnosed annually [3]. In addition, global mortality attributed to IHCC has recently increased [4].

IHCC diagnosis is complex, featuring a combination of appropriate clinical suspicion, imaging, endoscopy, and cytopathological examination. However, the fact that tumors are often detected in the late stages coupled with the poor prognosis of this disease, render it essential to identify biomarkers that allow early diagnosis. Carbohydrate antigen 19-9 (CA19-9), the most commonly used serum marker in clinical practice, is a sialylated Lewis blood group antigen than can be targeted by monoclonal antibodies. It was described in 1979 as a tumor-associated antigen in a colorectal cancer cell line [5]. CA19-9 measurements exhibit wide variation in sensitivity (50–90%) and specificity (54–98%), often being elevated in patients with benign biliary disease and/or cholangitis; the levels fall after relief of any biliary obstruction or sepsis [6, 7]. Despite widespread use of serum CA19-9 levels in IHCC patients, their prognostic role is less clear. Cytokeratins (CKs) are intermediate filaments that are essential for cytoskeleton formation in epithelial cells. Twenty different CK polypeptides have been identified, and CK-19 is associated with cholangiocarcinoma. It appears that CK-19 is more specific for IHCC than for other tumor types, but its sensitivity for tumor diagnosis is low [8].

Recently, we analyzed metabolites that were detected as low-mass ions by mass spectrometry (MS), and reported the metabolic profiles of biofluids (including urine and serum) of cancer patients. The data were useful for the early cancer diagnosis and prediction of prognosis [9–15]. Here, we described the MS-based metabolic profile of serum from IHCC patients, and explored the clinical significance of the upregulation of N’-methyl-2-pyridone-5-carboxamide levels in these patients.

## RESULTS

### Patient characteristics

The baseline characteristics of all of the IHCC patients are shown in Table 1. The median age (range) of the patients was 60 years (31–91 years), most of whom were males (70.1%). Of the 87 patients, 43% had T1 disease and 37% had lymph node (LN) metastasis. In addition, 41 (51%) had high CA19-9 levels (>37 U/mL) and 18 (21%) had chronic hepatitis B; Common bile duct stones were found in 3 patients (3.5%), and *g*allbladder stones were exhibited in 7 patients (8.1%). Serum CA 19-9 level was not associated with recurrence-free survival (RFS), even at a very high cut-off of 1,000 U/mL (Table 2).

**Table 1:** Baseline characteristics of the total IHCC patients

Characteristics		Number (%)
Follow-up duration	Median (range)	82.09 (1.28–140.35)
Age	Median (range)	60 (31–91)
Sex	Male	61 (70.1)
T stage^*^	1	37 (42.5)
	2	21 (24.1)
	3	19 (21.8)
	4	10 (11.5)
N stage1	1	23 (36.5)
M stage	1	5 (5.8)
CA19-92	> 37 U/mL	41 (50.6)
Total bilirubin level	Median (range)	0.6 (0.2–12.2)
HBsAg	+	18 (20.7)
Anti-HBs	+	47 (54.0)
Anti-HBc3	+	68 (84.0)
Anti-HCV4	+	3 (3.5)
Liver cirrhosis	+	13 (14.9)
History of CBD stone	+	3 (3.5)
History of GB stone	+	7 (8.1)

^*^7th AJCC TNM staging system, missing data: 1; *n =* 24, 2; *n =* 6, 3; *n =* 6; 4; *n =* 1

Common bile duct stone, CBD; gallbladder, GB.

**Table 2:** CA 19-9 level and RFS

U/mL	Frequency (%)			Cox (univariable)	
	Total	No event	Event	HR (95% CI)	*p*-value
≤ 22.9^*^	31	12 (38.7)	19 (61.3)	1	
> 22.9^*^	50	9 (18.0)	41 (82.0)	1.50 (0.87–12.60)	0.1454
≤ 37	40	12 (30.0)	28 (70.0)	1	
> 37	41	9 (22.0)	32 (78.1)	1.30 (0.78–2.16)	0.3180
≤ 100	56	17 (30.4)	39 (69.6)	1	
> 100	25	4 (16.0)	21 (84.0)	1.53 (0.90–2.61)	0.1178
≤ 1000	72	20 (27.8)	52 (72.2)	1	
> 1000	9	1 (11.1)	8 (88.9)	2.04 (0.96–4.33)	0.0627

^*^The cutoff was determined to maximize the differences between groups in terms of the log-rank test statistic.

### Identification of six metabolites differentially expressed in the serum of IHCC patients

**Figure 1:**
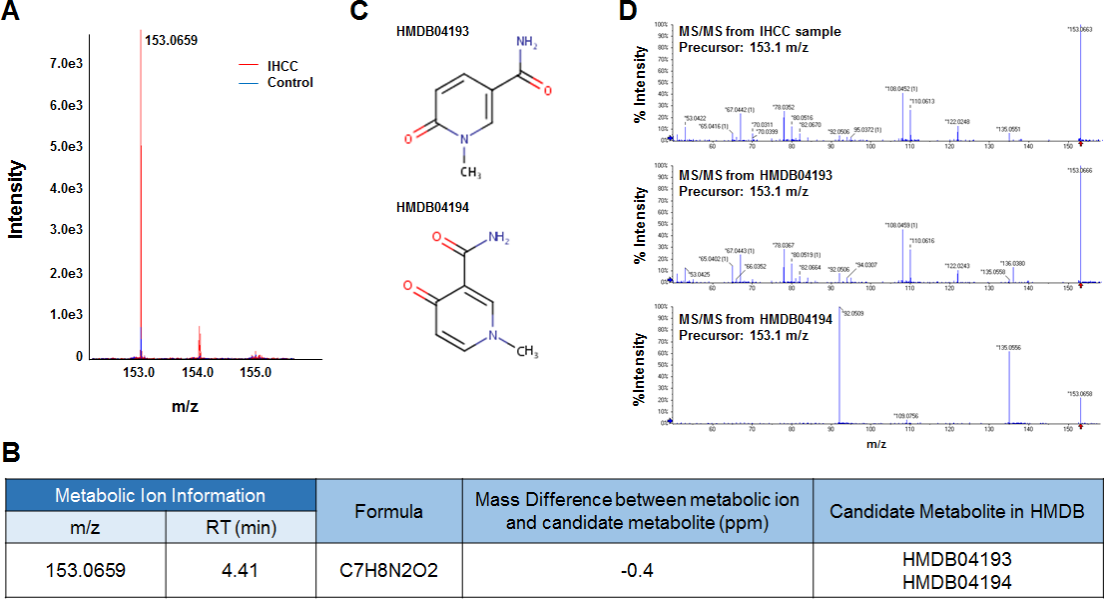
Identification of a metabolite ion of m/z 153.0659 with an RT of 4.41 min (**A**) The MS spectrum of the 153.0659 m/z ion. The intensity of the ion was greater in serum from IHCC patients (red peaks) than control subjects (blue peaks). (**B**) Candidate HMDB metabolites giving rise to the 153.0659 m/z ion. Based upon the MS information, the Formula Finder computational tools (SCIEX) selected two candidate metabolites as origins of the metabolic ion. (**C**) The structures of the candidate metabolites, HMDB04193 (2PY) and HMDB04194 (4PY). (**D**) MS/MS patterns at 153.0659 m/z from IHCC sera, 2PY, and 4PY. The MS/MS IHCC pattern was identical to that of 2PY.

**Figure 2:**
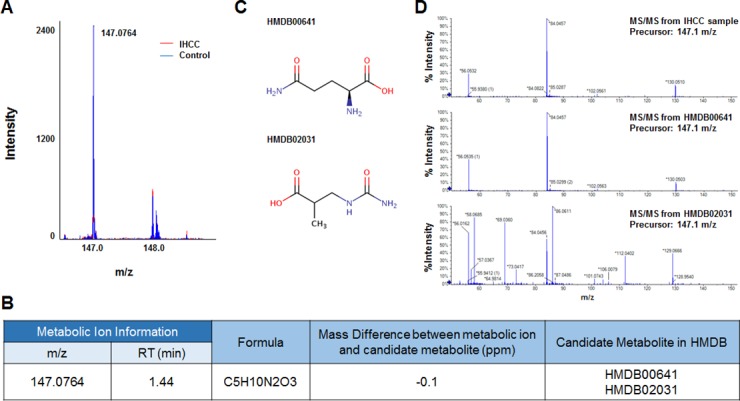
Identification of a metabolic ion of m/z 147.0764 with an RT of 1.44 min (**A**) The MS spectrum at 147.0764 m/z. The intensity of the ion was lower in serum from IHCC patients (red peaks) than control subjects (blue peaks). (**B**) Candidate HMBD metabolites yielding 147.0764 m/z ions. Two metabolic compounds were selected as candidates for the metabolic ion, HMDB00641 (L-glutamine) and HMDB02031 (ureidoisobutyric acid). (**C**) Structures of the candidate metabolites. (**D**) MS/MS patterns of the 147.0764 m/z ions from L-glutamine and ureidoisobutyric acid. The MS/MS pattern of the IHCC metabolic ion of 147.0764 m/z was identical to that of L-glutamine.

**Figure 3:**
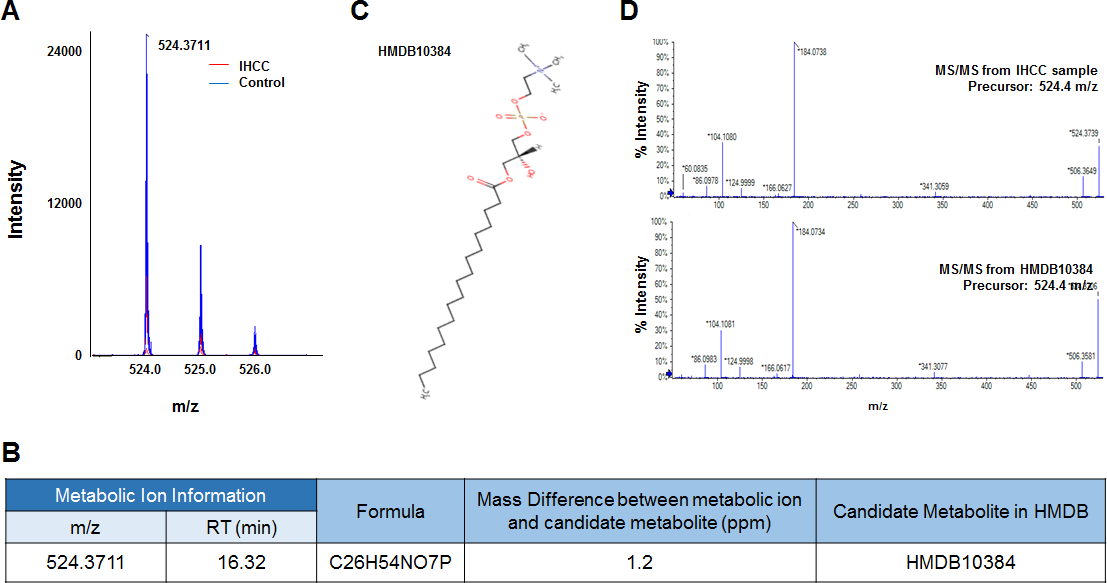
Identification of a metabolic ion of m/z 524.3711 with an RT of 16.32 min (**A**) The MS spectrum at 524.3711 m/z. The intensity of the ion was lower in serum from IHCC patients (red peaks) than control subjects (blue peaks). (**B**) A candidate HMDB metabolite giving rise to the 524.3711 m/z ion. Only one metabolic candidate was identified: HMDB10384 (LPC 18:0). (**C**) Structure of the candidate metabolite. (**D**) MS/MS pattern of the 524.3711 m/z ion from IHCC sera and LPC 18:0. The patterns were identical.

**Figure 4:**
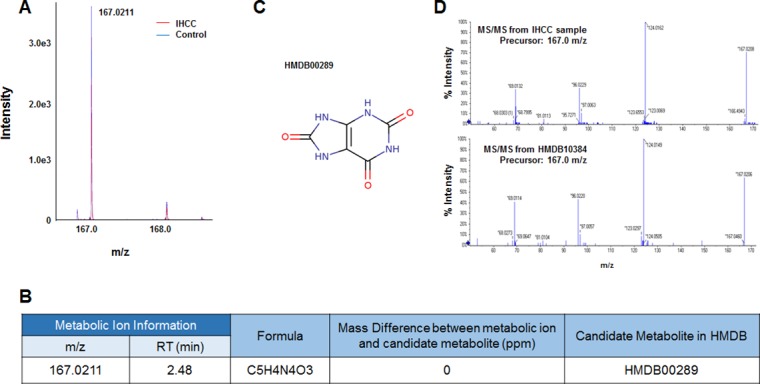
Identification of a metabolic ion of m/z 167.0211 m/z with an RT of 2.48 min (**A**) MS spectrum of the 167.0211 m/z in the negative detection mode. The intensity of the ion was slightly less in serum from IHCC patients (red peaks) compared to controls (blue peaks). (**B**) The candidate HDMB metabolite giving rise to the ion of 167.0211 m/z. Only one candidate was identified: HMDB00289 (uric acid). (**C**) Structure of the candidate metabolite. (**D**) The MS/MS patterns of the 167.0211 m/z from IHCC sera and uric acid. The patterns were identical.

Six metabolic ions that were differentially expressed in IHCC patients were identified with the aid of MarkerView software as described in the Materials and Methods section. The ion of 153.0659 m/z was attributable to one of two possible compounds: N’-methyl-2-pyridone-5-carboxamide (2PY) (HMDB04193) or N’-methyl-4-pyridone-5-carboxamide (4PY) (HMDB04194) (Figure 1A, 1B). Because the chemical structures of 2PY and 4PY are very similar (Figure 1C), the retention times (RTs) of both compounds were almost similar (~4.41 min) (Figure 1B). However, only 2PY exhibited a tandem mass spectrometry (MS/MS) pattern identical to that of the ion of 153.0659 m/z (Figure 1D). Similarly, two metabolites, L-glutamine (HMDB00641) and ureidoisobutyric acid (HMDB02031), were candidates for the origin of the metabolic ion of 147.0764 m/z with an RT of 1.44 min, but MS/MS analysis revealed that the material was in fact L-glutamine (Figure 2). Lysophosphatidylcholine (LPC) 18:0 was the only candidate for the origin of the metabolic ion of 524.3711 m/z with an RT of 16.32 min, and the MS/MS patterns of the two molecules were identical (Figure 3). The metabolic ion of (negative) 167.0211 m/z with an RT of 2.48 min was shown to have originated from uric acid (HMDB00289) (Figure 4). Two other metabolic ions (of 496.3398 m/z with an RT of 14.977 min and 768.8499 m/z with an RT 7.544 min) had been identified as LPC 16:0 [12] and fibrinopeptide A [9], respectively, in our previous studies.

### Quantification of the six differentially expressed metabolites in the serum from IHCC patients

**Figure 5:**
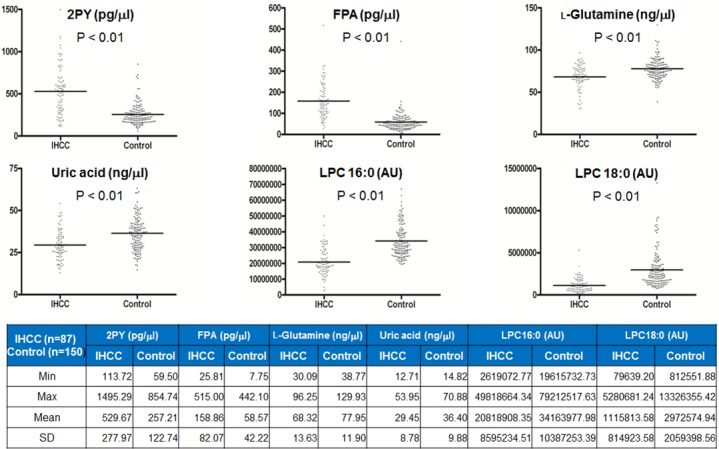
Quantification of six metabolites identified in sera of IHCC patients Six metabolites (2PY, FPA, L-glutamine, uric acid, LPC 16:0, and LPC 18:0) that were differentially expressed in sera from IHCC patients (compared to controls) were quantified using the MRM MS mode. 2PY and FPA levels were increased ca. 2- and 2.5-fold in sera from IHCC patients compared to controls, respectively (both *p* < 0.01). By contrast, L-glutamine, uric acid, LPC 16:0, and LPC 18:0 levels were reduced in sera from IHCC patients (all *p* < 0.01). AU: arbitrary unit.

The six metabolites described above, as revealed by MS/MS, were quantified in serum from 87 IHCC patients and 150 healthy controls using stable isotope-labeled compounds and internal standards as described in the Materials and Methods section. Of the six metabolites, 2PY and FPA were elevated by ca. 2- and 2.5-fold in serum from IHCC patients (2PY, 529.67 ± 277.97 pg/μL; FPA, 158.86 ± 82.07 pg/μL) compared to the controls (2PY, 257.21 ± 122.74 pg/μL; FPA, 58.57 ± 42.22 pg/μL) (both *p* < 0.01) (Figure 5). By contrast, L-glutamine, uric acid, LPC 16:0, and LPC 18:0 levels were reduced in the serum from IHCC patients (Figure 5). Although L-glutamine and uric acid levels significantly decreased, the decrease did not extend to fold changes (L-glutamine, IHCC 68.32 ± 13.63 ng/μL vs. control 77.95 ± 11.90 ng/μL; uric acid, IHCC 29.45 ± 8.78 ng/μL vs. control 36.40 ± 9.88 ng/μL) (both *p* < 0.01) (Figure 5). LPC 16:0 and LPC 18:0 were quantified by measuring their mass peak areas; the levels of both compounds were significantly reduced in the serum from IHCC patients (LPC 16:0, IHCC 20,818,908.35 ± 8,595,234.51 arbitrary units [AUs] vs. control 34,163,977.98 ± 10,387,253.39 AU; LPC 18:0, IHCC 1,115,813.58 ± 814,923.58 AU vs. control 2,972,574.94 ± 2,059,398.56 AU) (both *p* < 0.01) (Figure 5).

### The 2PY and LPC 16:0 levels were associated with RFS of IHCC patients

**Figure 6:**
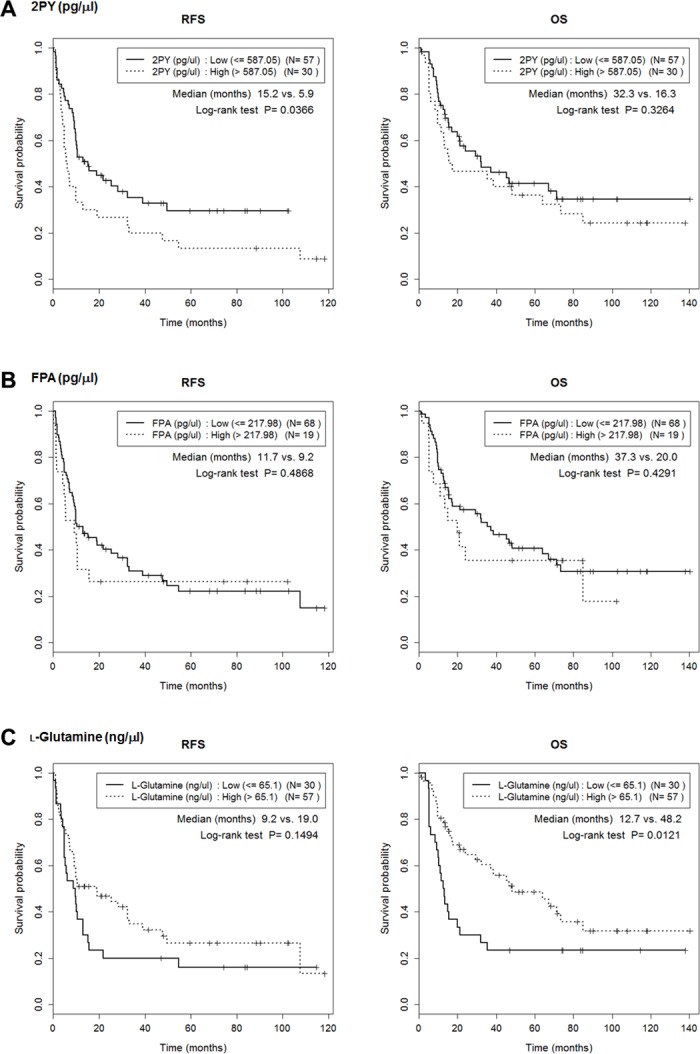

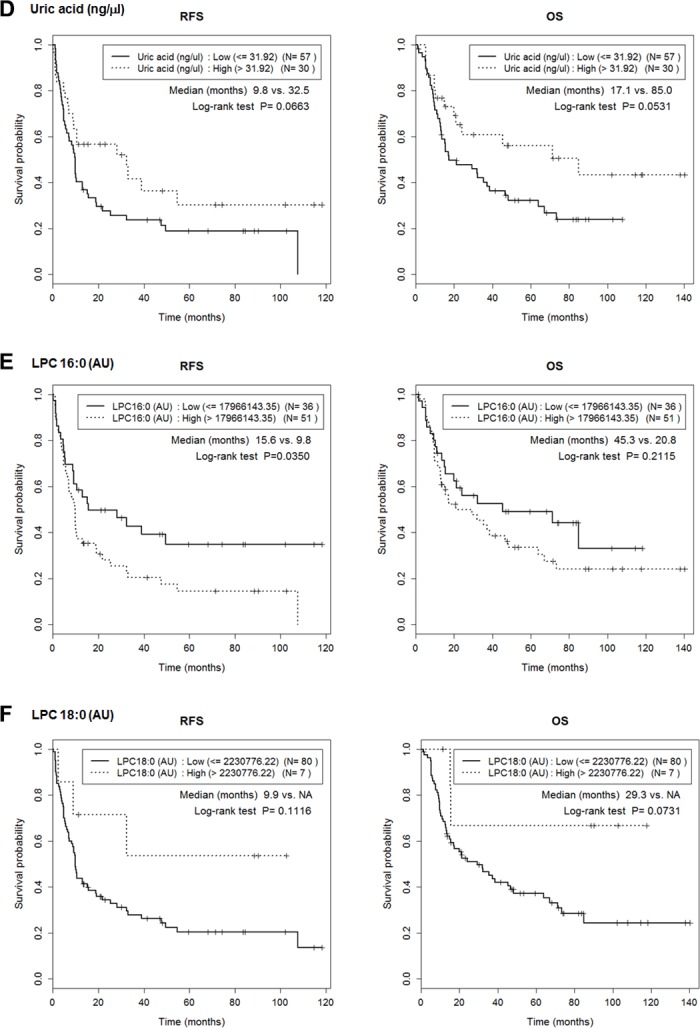
Lower 2PY and LPC16 levels were associated with better RFS Cutoffs for maximal prediction of high/low level RFSs were determined (Table 3). The Figure shows the associations between RFS or overall survival (OS) with the levels of the six metabolites, 2PY (**A**), FPA (**B**), L-glutamine (**C**), uric acid (**D**), LPC 16:0 (**E**) and LPC 18:0 (**F**). 2PY and LPC 16:0 showed significantly better RFS at low level than high level (*p* = 0.037 and *p* = 0.035, respectively). In addition, high-level L-glutamine was associated with a better OS (*p* = 0.0121).

We determined the cutoff values of the six metabolites that maximized the RFS differences (Table 3). No value was significantly associated with T or N stage (Supplementary Table 1), but the RFS of those with low and high 2PY levels significantly differed (median; 15.16 months vs. 5.90 months; *p* = 0.037) (Figure 6, Supplementary Table 2). 2PY and LPC 16:0 showed significantly better RFS at low level than high level (median; 15.62 months vs. 9.83 months; *p* = 0.035). IHCC patients with high (compared to low) levels of L-glutamine experienced better overall survival (OS) (median; 48.20 months vs. 12.71 months; *p* = 0.0121) (Figure 6).

**Table 3:** Candidate metabolic biomarkers

	Median	Cut-point
L-glutamine (ng/ul)	69.03	65.1
2PY (pg/ul)	520.36	587.05
FPA (pg/ul)	145.47	217.98
Uric acid (ng/ul)	28.17	31.92
LPC16 (Arbitrary Unit)	19214091.58	17966143.35
LPC18 (Arbitrary Unit)	928432.42	2230776.22

N’-methyl-2-pyridone-5-carboxamide, 2PY; fibrinopeptide A, FPA; lysophosphatidylcholine, LPC.

## DISCUSSION

We found that six metabolites (L-glutamine, LPC 16:0, LPC 18:0, 2PY, FPA, and uric acid) were candidate predictors of IHCC prognosis (Figures 1–4). Of these, the 2PY level was significantly increased in IHCC patients (*p* < 0.01) (Figure 5), and a high level significantly correlated with poor RFS (median; 15.16 months vs. 5.90 months, *p* = 0.037) (Figure 6). Nicotinamide, 2PY, and 4PY are metabolites of the intracellular co-enzyme nicotinamide adenine dinucleotide (NAD), and potentially inhibit poly(ADP-ribose)polymerase (PARP)-1, a DNA repair enzyme [16]. NAD catabolism in mammalian cells occurs principally via a reaction catalyzed by PARP, releasing nicotinamide, which then is predominantly metabolized to 2PY [16, 17]. PARP is involved in DNA repair, the cell stress response, and regulation of apoptosis. Therefore, NAD metabolites including 2PY may accumulate under diseased conditions, accelerating DNA damage and causing the retention of catabolic products [16, 17].

NAD metabolites may be particularly harmful in children (who exhibit more DNA turnover than adults); NAD metabolites accumulate in the plasma of children with chronic renal failure [18]. In addition, the level of 2PY, an end product of NAD degradation, increases in the serum of chronic renal failure patients, and is associated with the deterioration of kidney function and toxicities including significant PARP-1 inhibition. 2PY was also elevated in the plasma of uremic patients [19]. Accumulation of 2PY in erythrocytes was positively correlated with the extent of renal failure [20]. Therefore, 2PY may be a novel toxin that plays a significant role in the development of uremic toxemia, particularly by acting as a PARP inhibitor [16, 17, 21]. By contrast, one report found that 2PY protected endothelial cells from oxidative stress injury by inhibiting PARP and reducing NAD depletion. This maintained cellular energetics and high ATP levels [22].

Apart from the reported association with renal failure, the effects of 2PY on other diseases have received little attention. As liver cirrhosis progresses, the extent of nicotinamide methylation and urinary N-methylnicotinamide and 2-pyridone-5-carboxamide levels gradually rise [23]. Hypermethylation might protect against the toxic effects of intracellular nicotinamide attributable to cirrhosis-associated catabolism [23]. Further work is needed to explain why 2PY levels increase in the sera from IHCC patients; it may be a useful biomarker not only when screening for IHCC but also for predicting disease prognosis.

Unlike 2PY, the levels of LPC 16:0 and LPC 18:0 decreased in serum from IHCC patients (*p* < 0.01) (Figure 5), and low levels of LPC 16:0 (compared to high levels) significantly correlated with an improved RFS (median; 15.62 months vs. 9.83 months, *p* = 0.035) (Figure 6). Lysophosphatidic acid (LPA; monoacyl-glycerol-3-phosphate) serves as a mitogen and a stimulator of motility in many cell types [24], acting in either an autocrine or paracrine fashion via G protein-coupled receptors. LPA is involved in many physiological and pathological processes, including cancer [25]. LPA enhances tumor growth, metastasis, and chemoresistance [26]. LPA is derived from lysophosphatidylcholine (LPC) via the action of a secreted phospholipase termed autotaxin (ATX), which was originally identified as an “autocrine motility factor” of tumor cells [24]. As ATX promotes both tumor formation and angiogenesis [24], and as ATX levels are elevated in several human malignancies [24, 27], many researchers believe that ATX may be a promising therapeutic target in patients with chronic inflammation and cancer [25, 26, 28–30].

Similar to LPA and ATX, reduced LPC levels have been reported in the blood and tissue samples of patients with many types of cancer (serum from ovarian cancer patients [12]; and tissues from gastric [31], prostate [32], and liver [33] cancer patients). Higher LPC 18:0 plasma levels were associated with lower risks of breast, prostate, and colorectal cancers [34]. The plasma proportions of 18:1-LPC or 18:2-LPC (in terms of total saturated LPC levels), either individually or combined, are potential biomarkers for CRC [35]. Reduced LPC levels appear to be strongly associated with the activity of lysophosphatidylcholine acyltransferase 1 (LPCAT1), which converts LPC into phosphatidylcholine (PC). LPCAT1 was highly expressed in gastric cancer lesions compared to non-neoplastic mucosal tissues, predominantly in patients with differentiated adenocarcinomas [31]. PC and LPC 16:0 levels were higher and lower, respectively, in gastric cancer tissues of such patients [31]. LPCAT1 overexpression has been noted in many other types of cancer including breast [36] and colorectal [37] cancers, and the level has often been considered clinically relevant in tumorigenesis [31, 36, 38–40].

Significantly lower levels of LPC were evident in the bile of patients with biliary tract cancer compared to those with benign biliary tract disease [41]. Therefore, LPC may be a novel biomarker allowing early detection of biliary tract cancer [30]. Recent studies have shown that LPC may trigger cholangiocyte senescence, thereby potentially contributing to the pathogenic development of biliary tract cancer [42]. LPC also inhibited cholangiocyte apoptosis by inducing COX-2 expression via a Raf-1-dependent mechanism [43]. Such anti-apoptotic effects of LPC may play roles in biliary tract carcinogenesis in patients with anomalous pancreaticobiliary ductal junctions. Inhibition of these LPC actions may be a valuable chemopreventative strategy [43]. These studies support our finding that LPC 16:0 levels were reduced in the serum from IHCC patients, and strongly suggest that the LPC 16:0 levels may be metabolically significant in terms of IHCC prognosis.

We identified six metabolites (including 2PY and LPC 16:0) that were candidate metabolic biomarkers of IHCC. Of these, the 2PY and LPC 16:0 levels may be prognostic. Integration of information obtained upon quantification of the six individual metabolites identified in this study will lay a strong foundation for future validation of our results.

## MATERIALS AND METHODS

### Chemicals and reagents

LC-MS-grade acetonitrile and 0.1% (v/v) formic acid (FA) in water were purchased from Thermo Scientific (Waltham, MA, USA). LC-MS-grade water was from Fisher Scientific (Hampton, NH, USA) and methanol was from Honeywell Burdick & Jackson (Morris Plains, NJ, USA). L-glutamine, human FPA, uric acid, bovine serum albumin, and dichloromethane were obtained from Sigma-Aldrich (St. Louis, MO, USA). LPC 16:0 and LPC 18:0 were the products of Avanti Polar Lipids (Alabaster, Al, USA) and 2PY was obtained from Santa Cruz Biotechnology (Dallas, TX, USA). The internal standards were L-glutamine-^13^C_5_, [Tyr°]-human FPA, uric acid-1,3–^15^N_2_ (from Sigma-Aldrich) and 4PY (from Carbosynth [Compton, UK]). APCI-positive and ACPI-negative calibration solutions were the products of SCIEX (Framingham, MA, USA).

### Serum extraction for metabolite profiling

A total of 237 serum samples from IHCC patients (*n* = 87) and healthy controls (*n* = 150) were extracted using the modified Bligh and Dyer method (14). In brief, 50 μL serum was added to 1 mL water. After vortexing, 2 mL MeOH and 0.9 mL dichloromethane were added. After vortexing and placing on ice for 30 min, 1 mL water and 0.9 mL dichloromethane were added, and the mixture was centrifuged (1,500 rpm, 10 min, room temperature). The supernatant was dried under an N_2_ stream and subjected to MS analysis.

### Serum metabolite profiling

The dried samples were reconstituted in 0.1% (v/v) FA and subjected to liquid chromatography (LC)-MS/MS using a Shimadzu Nexera X2 system (Shimadzu, Chiyoda-ku, Tokyo, Japan) coupled to a SCIEX Triple TOF 5600+ module (SCIEX) equipped at the front end with a DuoSpray ion source. For ultra-high-performance LC, samples were loaded into Atlantis T3 sentry guard cartridges (3 μm, 2.1 mm × 10 mm; Waters Corporation [Milford, MA, USA]) and separation proceeded on an Atlantis T3 column (3 μm, 2.1 mm × 100 mm; Waters). The MS system was set to perform one full scan (50 to 1,200 m/z) followed by MS/MS of the 10 most abundant parent ions (mass tolerance, 50 mDa; collision energy, 35%).

### Selection and identification of metabolite ions

LC/MS peak lists (.peaks files) were created for all of the samples (.wiff files) using MarkerView software running under the following constraints: minimum retention time, 0.00 min; subtraction offset, 10 scans; subtraction multiplication factor, 1.3; noise threshold, 10; minimum spectral peak width, 10 ppm; and minimum RT peak width, 5 scans. Next, a peak table was created by simultaneously importing the LC/MS peak lists for all of the samples. The parameters for the second process were as follows: RT tolerance, 0.01 min; mass tolerance, 10.0 ppm; intensity threshold, 10; and maximum number of peaks, 20,000. Areas were derived using raw data, and not using the original peak findings, as suggested by the reference manual. The resulting peak table was normalized using the “total area sums” method and the data converted into common logarithms. The MS and MS/MS spectra were submitted to the Formula Finder computational tools (SCIEX) that propose probable elemental compositions within a specified mass tolerance of a given mass-to-charge ratio (m/z), using PeakView software (SCIEX). By interrogating the HMDB metabolite databases, the specific compounds giving rise to the observed m/z ions were identified, and listed in rank order based on the MS and MS/MS data. Proteomic MS/MS analyses were performed with the aid of ProteinPilot software (SCIEX).

### Quantification of the six selected metabolites

For quantitative analyses, serum samples were mixed with L-glutamine-^13^C_5_, [Tyr]-human FPA, uric acid-1,3–^15^N_2_, LPC 16:0-d31, and 4PY (internal standards). Varying amounts of standards were spiked into bovine serum albumin solutions in the presence of the internal standard, and extracted. MS analyses were performed using a Triple TOF 5600+ system (SCIEX) fitted with an analyst data acquisition module. The positive ion multiple reaction monitoring (MRM) mode was used for quantitative analysis of L-glutamine, LPC 16:0, LPC 18:0, 2PY, and FPA. Sample extracts (5 μL) were loaded via a Nexera X2 LC system (Shimadzu) onto a three-step linear gradient (solvent A, 0.1% [v/v] FA in water; solvent B, 100% ACN; 1% solvent B for 1.5 min, 1–25% B for 4.5 min, 25–45% B for 2 min, 45–90% B for 2 min, 90% B for 4 min, 90–1% B for 0.5 min, and 1% B for 5.5 min). The negative ion MRM mode was used for quantitative analysis of uric acid levels. LC separations (10 min/sample) were performed using a one-step linear gradient (solvent A, 0.1% [v/v] FA in water; solvent B, 100% ACN; 1% solvent B for 1.5 min, 1–90% B for 2.5 min, 90% B for 2 min, 90–1% B for 0.5 min, and 1% B for 3.5 min). The six metabolites were quantified with the aid of MultiQuant software (SCIEX).

### Statistical analysis

The Cox proportional hazard model was used to test for difference in RFS between two groups dichotomized according to CA 19-9 level. The levels of continuous biomarkers were dichotomized based on cutoffs maximizing the differences between groups in terms of the log-rank test statistic [44]. The RFS differences between the two groups were compared using the log-rank test, and survival was estimated by drawing Kaplan–Meier curves.

## SUPPLEMENTARY MATERIALS TABLES




