# Supplementary material for: Reduced levels of N’-methyl-2-pyridone-5-carboxamide and lysophosphatidylcholine 16:0 in the serum of patients with intrahepatic cholangiocarcinoma, and the correlation with recurrence-free survival
Source: Oncotarget. 2017 Nov 22;8(68):112598–609. doi: 10.18632/oncotarget.22607 (PMC5762534; doi:10.18632/oncotarget.22607)
Supplement: Supplementary file 1 [file oncotarget-08-112598-s001.pdf]

Reduced levels of N'-methyl-2-pyridone-5-carboxamide and lysophosphatidylcholine 16:0 in the serum of patients with intrahepatic cholangiocarcinoma, and the correlation with recurrence-free survival

SUPPLEMENTARY MATERIALS

Supplementary Table 1: No values of the candidate metabolites were significantly associated with T or N stage. See_Supplementary_Table 1

Supplementary Table 2: 2PY and LPC16 level was associated with RFS

		Total	RFS (event)		*p*-value
			No	Yes	
		(*N* = 87)	(*N* = 23)	(*N* = 64)	
L-Glutamine (ng/ul)	Low	30	5 (16.67)	25 (83.33)	0.1494
	High	57	18 (31.58)	39 (68.42)	
2PY (pg/ul)	Low	57	20 (35.09)	37 (64.91)	0.0366
	High	30	3 (10.00)	27 (90.00)	
FPA (pg/ul)	Low	68	18 (26.47)	50 (73.53)	0.4868
	High	19	5 (26.32)	14 (73.68)	
Uric acid (ng/ul)	Low	57	11 (19.30)	46 (80.70)	0.0663
	High	30	12 (40.00)	18 (60.00)	
LPC16:0 (AU)	Low	36	14 (38.89)	22 (61.11)	0.035
	High	51	9 (17.65)	42 (82.35)	
LPC18:0 (AU)	Low	80	19 (23.75)	61 (76.25)	0.1116
	High	7	4 (57.14)	3 (42.86)	

Recurrence-free survival, RFS; arbitrary unit, AU.
