# Supplementary material for: Reduced levels of N’-methyl-2-pyridone-5-carboxamide and lysophosphatidylcholine 16:0 in the serum of patients with intrahepatic cholangiocarcinoma, and the correlation with recurrence-free survival
Source: Oncotarget. 2017 Nov 22;8(68):112598–609. doi: 10.18632/oncotarget.22607 (PMC5762534; doi:10.18632/oncotarget.22607)
Supplement: Supplementary file 2 [file oncotarget-08-112598-s002.docx]

**Supplementary Table 1.** No values of the candidate metabolites were significantly associated with T or N stage.

| a. L-Glutamine (ng/ul) | |  |  |  |  |
| --- | --- | --- | --- | --- | --- |
|  | | N | L-Glutamine (ng/ul) | p-value |  |
|  |  |  | median (range) |  |  |
| T stage | 1 | 37 (42.5) | 68.6 (30.09-89.4) | 0.300 | ‡ |
|  | 2 | 21 (24.1) | 75.4 (31.03-96.3) |  |  |
|  | 3 | 19 (21.8) | 69 (40.55-85.1) |  |  |
|  | 4 | 10 (11.5) | 63.2 (34.92-84) |  |  |
|  |  |  |  |  |  |
| N stage (miss=24) | 0 | 40 (63.5) | 70.4 (31.03-96.3) | 0.733 | * |
|  | 1 | 23 (36.5) | 67.9 (30.09-87.6) |  |  |
|  |  |  |  |  |  |
| CA19-9 (miss=6) | <= 37 | 40 (49.4) | 70.8 (31.03-89.4) | 0.020 | * |
|  | > 37 | 41 (50.6) | 65.5 (30.09-96.3) |  |  |

| b. 2PY (pg/ul) |  |  |  |  |
| --- | --- | --- | --- | --- |
|  | | N | 2PY (pg/ul) | p-value |
|  |  |  | median (range) |  |
| T stage | 1 | 37 (42.5) | 531.7 (113.7-1178.8) | 0.982 |
|  | 2 | 21 (24.1) | 510.4 (117.6-1495.3) |  |
|  | 3 | 19 (21.8) | 529.4 (120.6-1077.4) |  |
|  | 4 | 10 (11.5) | 539.8 (170.2-1042.1) |  |
|  |  |  |  |  |
| N stage (miss=24) | 0 | 40 (63.5) | 524.9 (117.6-1495.3) | 0.249 |
|  | 1 | 23 (36.5) | 587.1 (183.8-1178.8) |  |
|  |  |  |  |  |
| CA19-9 (miss=6) | <= 37 | 40 (49.4) | 536.4 (117.6-1495.3) | 0.774 |
|  | > 37 | 41 (50.6) | 487.3 (113.7-1077.4) |  |

| c. FPA (pg/ul) |  |  |  |  |
| --- | --- | --- | --- | --- |
|  | | N | FPA (pg/ul) | p-value |
|  |  |  | median (range) |  |
| T stage | 1 | 37 (42.5) | 145.5 (34.65-326.5) | 0.811 |
|  | 2 | 21 (24.1) | 139.5 (55.82-515) |  |
|  | 3 | 19 (21.8) | 142.6 (25.81-292.1) |  |
|  | 4 | 10 (11.5) | 184.3 (80.95-290.8) |  |
|  |  |  |  |  |
| N stage (miss=24) | 0 | 40 (63.5) | 143.1 (52.59-326.5) | 0.983 |
|  | 1 | 23 (36.5) | 154.2 (34.65-308.4) |  |
|  |  |  |  |  |
| CA19-9 (miss=6) | <= 37 | 40 (49.4) | 119.2 (25.81-326.5) | 0.105 |
|  | > 37 | 41 (50.6) | 158.7 (44.06-515) |  |

| d. Uric acid (ng/ul) |  |  |  |  |
| --- | --- | --- | --- | --- |
|  | | N | UA (ng/ul) | p-value |
|  |  |  | median (range) |  |
| T stage | 1 | 37 (42.5) | 29.6 (15.52-48.6) | 0.812 |
|  | 2 | 21 (24.1) | 27.5 (15.06-54) |  |
|  | 3 | 19 (21.8) | 27.9 (17.35-43.3) |  |
|  | 4 | 10 (11.5) | 29.3 (12.71-36.7) |  |
|  |  |  |  |  |
| N stage (miss=24) | 0 | 40 (63.5) | 27.9 (12.71-48.6) | 0.943 |
|  | 1 | 23 (36.5) | 28.2 (20.29-46.4) |  |
|  |  |  |  |  |
| CA19-9 (miss=6) | <= 37 | 40 (49.4) | 28.8 (15.06-54) | 0.778 |
|  | > 37 | 41 (50.6) | 27.5 (12.71-48.2) |  |

| e. LPC16:0  (arbitrary unit) |  |  |  |  |  |
| --- | --- | --- | --- | --- | --- |
|  | | N | LPC16:0 | p-value |  |
|  |  |  | median (range) |  |  |
| T stage | 1 | 37 (42.5) | 18113830.9 (2619072.77-43887943.5) | 0.807 | ‡ |
|  | 2 | 21 (24.1) | 19812145.4 (4775732.6-49818664.3) |  |  |
|  | 3 | 19 (21.8) | 20100671.5 (13115304.75-34915682.2) |  |  |
|  | 4 | 10 (11.5) | 19188966.3 (11243012.78-37499802.7) |  |  |
|  |  |  |  |  |  |
| N stage (miss=24) | 0 | 40 (63.5) | 19695458.2 (3263221.96-49818664.3) | 0.368 | * |
|  | 1 | 23 (36.5) | 20361303.4 (9340080.08-37499802.7) |  |  |
|  |  |  |  |  |  |
| CA19-9 (miss=6) | <= 37 | 40 (49.4) | 20432652.2 (2619072.77-49818664.3) | 0.087 | * |
|  | > 37 | 41 (50.6) | 18080935.4 (4775732.6-43887943.5) |  |  |

| f. LPC18:0  (arbitrary unit) |  |  |  |  |
| --- | --- | --- | --- | --- |
|  | | N | LPC18:0 | p-value |
|  |  |  | median (range) |  |
| T stage | 1 | 37 (42.5) | 928432.4 (79639.2-5280681.2) | 0.754 |
|  | 2 | 21 (24.1) | 1285285 (120795.4-3380678.3) |  |
|  | 3 | 19 (21.8) | 819890.2 (360802.99-2430640.6) |  |
|  | 4 | 10 (11.5) | 729669.4 (286540.99-2085807) |  |
|  |  |  |  |  |
| N stage (miss=24) | 0 | 40 (63.5) | 899328.4 (120795.4-5280681.2) | 0.793 |
|  | 1 | 23 (36.5) | 1023346.7 (314413.79-2069676.9) |  |
|  |  |  |  |  |
| CA19-9 (miss=6) | <= 37 | 40 (49.4) | 1012098.8 (79639.2-2630396.7) | 0.084 |
|  | > 37 | 41 (50.6) | 725291.5 (120795.4-5280681.2) |  |
| median (range; min-max) | |  |  |  |
| p-value* was calculated using Wilcoxon rank-sum test. | | | |  |
| p-valeu‡ was calculated using Kruskal-wallis test. | | | |  |
